# NLRP3 Inflammasome Expression and Signaling in Human Diabetic Wounds and in High Glucose Induced Macrophages

**DOI:** 10.1155/2017/5281358

**Published:** 2017-01-10

**Authors:** Xiaotian Zhang, Jiezhi Dai, Li Li, Hua Chen, Yimin Chai

**Affiliations:** ^1^Department of Orthopedic Surgery, Shanghai Sixth People's Hospital, Jiao Tong University, Shanghai, China; ^2^Department of Orthopedic Surgery, Shanghai First People's Hospital, Jiao Tong University, Shanghai, China

## Abstract

*Introduction.* To investigate the contribution and mechanism of NLRP3 inflammasome expression in human wounds in diabetes mellitus and in high glucose induced macrophages.* Methods.* In the present study, we compared the expression of NLRP3 inflammasome in debridement wound tissue from diabetic and nondiabetic patients. We also examined whether high glucose induces NLRP3 inflammasome expression in cultures THP-1-derived macrophages and the influence on IL-1*β* expression.* Results.* The expressions of NLRP3, caspase1, and IL-1*β*, at both the mRNA and protein level, were significantly higher in wounds of diabetic patients compared with nondiabetic wounds (*P* < 0.05). High glucose induced a significant increase in NLRP3 inflammasome and IL-1*β* expression in THP-1-derived macrophages. M1 macrophage surface marker with CCR7 was significantly upregulated after high glucose stimulation. SiRNA-mediated silencing of NLRP3 expression downregulates the expression of IL-1*β*.* Conclusion.* The higher expression of NLRP3, caspase1, and secretion of IL-1*β*, signaling, and activation might contribute to the hyperinflammation in the human diabetic wound and in high glucose induced macrophages. It may be a novel target to treat the DM patients with chronic wound.

## 1. Introduction

Diabetes mellitus affects more than 170 million people worldwide with the number expected to increase [1]. Impaired wound healing is a major complication and chronically contributes to a poor quality of life associated with pain, suffering, and disability [2]. Enhanced activation of inflammatory pathology accumulation clearly contributes to the healing impairment. IL-1*β* is a proinflammatory cytokine that is produced by various cells such as neutrophils, macrophages, fibroblasts, and keratinocytes. Excessive IL-1*β* production is closely linked to chronic inflammatory disease, including diabetes, atherosclerosis, and gout [3]. An important evidence of IL-1*β* is reported in mice showing that IL-1*β* has a negative effect on angiogenesis in wound healing [4, 5]. NLRP3 is one of the NLR family members. Once activated, procaspase1 is recruited to the NLRP3 inflammasome and cleaved to produce active caspase1 and then cleaves and activates the potent proinflammatory cytokines IL-1*β* and IL-18. Caspase1 is key constituent of this inflammasome together with NLRP3 and an adapter protein termed apoptosis-associated speck-like protein containing a caspase recruitment domain (ASC) [6].

The stimuli to activate the NLRP3 inflammasome during infection include bacterial, viral, and fungal pathogens [7]. Moreover, the activation of NLRP3 inflammasome is induced by endogenous and exogenous danger signals, such as lipopolysaccharide (LPS) and high glucose (HG). During impaired healing associated with diabetes, wounds display prolonged accumulation of macrophages associated with elevated levels of proinflammatory cytokines [8]. However, the role of the NLRP3 inflammasome in high glucose induced macrophages remains unclear.

Recent studies have suggested NLRP3 inflammasome/IL-1*β* pathway plays a critical role in the pathogenesis of type 2 diabetes mellitus [9], and other evidences have previously shown that sustained NLRP3 inflammasome activity contributes to impairing wound healing in diabetic mice [10]. However, little is known about the role of the NLRP3 inflammasome in human diabetic wounds. Thus, in the present study, we examined NLRP3 inflammasome expression in human diabetic wounds and in high glucose induced macrophages.

## 2. Materials and Methods

### 2.1. Patients

A chronic wound is a wound that does not heal in an orderly set of stages and in a predictable amount of time the way most wounds do; wounds that do not heal within three months are often considered chronic [11]. In this study, we included type 2 diabetes with chronic wounds located anywhere on the foot and non-DM patients (controls) with a leg wound lasting for at least three months. We collected wound tissue from diabetic (*n* = 6) and nondiabetic wound (*n* = 6) during initial debridement as part of standard of care. Patients' evaluation included a medical history, physical examination, and wound site measurements (location, size, and clinical infection). Serum glucose and HbA1c levels were extracted from patients.

Inclusion criteria included age 18 and older; ulcer size >2 cm^2^ and <25 cm^2^; ulcer duration more than three months; no clinical signs of infection; and adequate circulation to the affected extremity.

Exclusion criteria were chronic wound caused by pressure ulcer, vasculitis, pyoderma gangrenosum, and diseases that cause ischemia; osteomyelitis or index ulcer probing to bone; currently receiving radiation or chemotherapy; known or suspect malignancy of current ulcer. This study was approved by the Ethic Review Board of Shanghai Six People's Hospital affiliated to Shanghai Jiao Tong University. Written informed consent was obtained from all of the enrolled participants.

### 2.2. Cell Culture, Differentiation, and High Glucose Stimulation

The THP-1 cell line was obtained from the Cell Bank of the Chinese Academy of Sciences (Shanghai, China) and maintained at 2–10 × 10^5^ cells/mL in the RPMI 1640 medium supplemented with 10% fetal bovine serum, 100 U/mL penicillin, and 0.1 mg/mL streptomycin at 37°C with 5% CO_2_. The differentiation of THP-1 cells into macrophage s was induced with phorbol myristate acetate (PMA) for 72 h. After washing with phosphate-buffered saline (PBS), the THP-1-derived macrophages were exposed to a high glucose environment. Control group (NC) were incubated in a medium containing 10% FBS, 2 mmol L-glutamine, 50 *μ*g/ml gentamicin, and 5.5 mmol glucose for 48 h designed to resemble normal glucose levels observed in healthy subjects. High glucose group (HG) were incubated in a medium containing 10% FBS, 2 mmol L-glutamine, 50 *μ*g/ml gentamicin, and 30 mmol glucose for 48 h.

### 2.3. Collection of Wound Tissue

During a sharp debridement, biopsies were taken from tissue located near the center of the wound. Immediately, it was frozen in liquid nitrogen for mRNA and protein analyses.

### 2.4. RNA Analysis

Total RNA was isolated from all the snap frozen wound tissue and macrophages with Trizol (Invitrogen). The first strand cDNA was synthesized with 2 *μ*g total RNA using a reverse transcriptase kit from (Promega). Quantitative PCR was performed in 20 *μ*l total reaction mix using PCR master mix (Life Technology) on an ABI ViiA7 (Life Technology) following the manufacturer's cycling parameters. Relative gene expression was determined using the 2^−ΔCT^ method and *β*-actin was used as endogenous control.

### 2.5. Western Blot

Expression of NLRP3 proteins was evaluated by Western blot. Primary antibodies for NLRP3 were purchased from Cell Signaling (Danvers, MA, USA). Secondary peroxidase conjugated antibodies were obtained by Abcam (Cambridge, UK). The protein signals were visualized by chemiluminescence (ECL, Promega), quantified by scanning densitometry, and were expressed as integrated intensity, relative to *β*-actin (Cell Signaling), measured on stripped blots.

### 2.6. Elisa

IL-1*β* was measured in the supernatants of wound homogenates or cell culture medium using human-specific ELISA assay kits (from Anogen and RayBiotech, resp.) and following the manufacturer's instructions. IL-1*β* levels were expressed as pg/mL of wound lysate.

### 2.7. Immunofluorescence

Sections were incubated overnight with primary antibodies against CD68 and NLRP3. Sections were then incubated with FITC- and tetramethylrhodamine isothiocyanate-conjugated isotype specific secondary antibodies.

### 2.8. Characterization of Cell Surface Markers by Flow Cytometry

After collecting macrophages, 5 × 10^5^ cells were resuspended in 50 *μ*L of sterile PBS. Then, cells were simultaneously incubated with anti-CCR7 2 *μ*g or anti-206 6 *μ*g for 45 min on ice, respectively. After incubation and three rounds of wash in the same buffer, cells were incubated with FITC anti-mouse antibody for 25 min on ice. The samples were analyzed using BD FACSCalibur and the results were analyzed using FlowJo software.

### 2.9. Data Analysis

Data are presented as mean ± SD. Comparisons between different groups were analyzed by one-way ANOVA test. A *P* value < 0.05 was considered significant. All statistical analyses were performed using GraphPad Prism software.

## 3. Results

Six patients had diabetes mellitus for >5 years (mean 2 h glucose of 13.7 ± 2.2 mg/dL and HbA1c of 7.8%  ±  1.2%) with six non-DM patients on routine standard care for a chronic wound of at least three months of duration and showed no signs of clinical infection. All the clinical data and laboratory data were documented in Tables 1 and 2. In diabetic wound group, two were in ankle, one was in midfoot, and three were in forefoot. None of them was in plantar surface. Patient did not report the existence of other diabetic complications, such as peripheral neuropathy, nephropathy, retinopathy, and vascular disease. In control group, patients did not suffer from other diseases.

### 3.1. Expression of NLRP3, Caspase1, and IL-1*β* in Human Diabetic Wound

**Figure 1:**
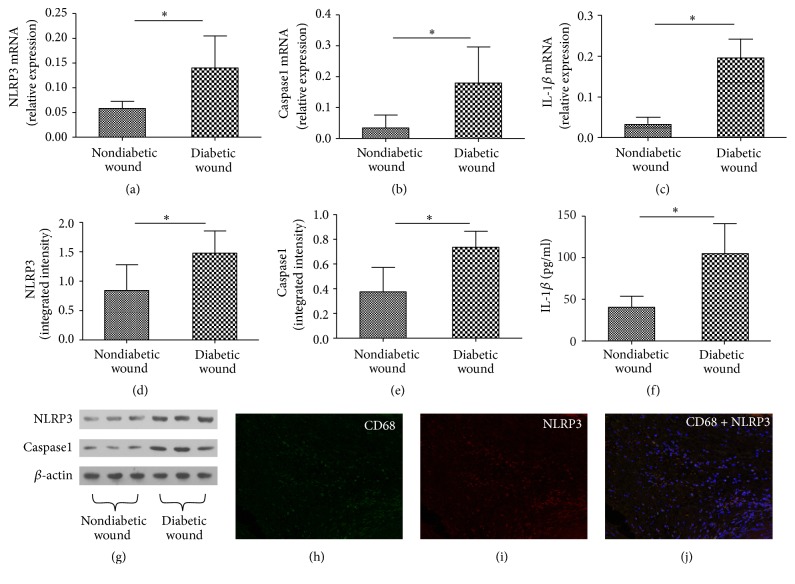
mRNA and protein expression of NLRP3, caspase1, and IL-1*β* in diabetic wound and nondiabetic wound. RNA was measured by real-time PCR (a, b, and c) and proteins were measured by Western blot (d, e, and g) or Elisa (f), respectively. Wound biopsy immunostained for NLRP3 and the macrophages marker CD68 (h, i, and j). Values are mean ± SD; ^*∗*^*P* < 0.05.

Total RNA was isolated from all the snap frozen wound tissue of patients with diabetic wound or nondiabetic wound for this analysis of NLRP3, caspase1, and IL-1*β* mRNA expression. A significantly higher level of NLRP3, caspase1, and IL-1*β* was found in patients of diabetic wound compared to nondiabetic wound (*P* < 0.05) (Figures 1(a), 1(b), and 1(c)). Consistent with mRNA expression pattern, a similar expression profile of protein expression of NLRP3, caspase1, and IL-1*β* was observed between diabetic wound and nondiabetic wound (Figures 1(d)–1(g)).

### 3.2. Wound Biopsy Immunostained for NLRP3 and the Macrophages Marker CD68

Immunofluorescent staining for NLRP3 was performed in chronic wound paraffin section. It confirmed protein expression of the NLRP3, in the majority of CD68^+^ wound macrophages (Figures 1(h), 1(i), and 1(j)).

### 3.3. High Glucose Induces the Production of NLRP3, Caspase1, and IL-1*β* in Macrophages

With the confirmed previous reports that NLRP3 inflammation signaling pathway is responsive to high glucose in macrophages, we treated the human THP-1-derived macrophages for 3 d with 30 mmol/L glucose.

**Figure 2:**
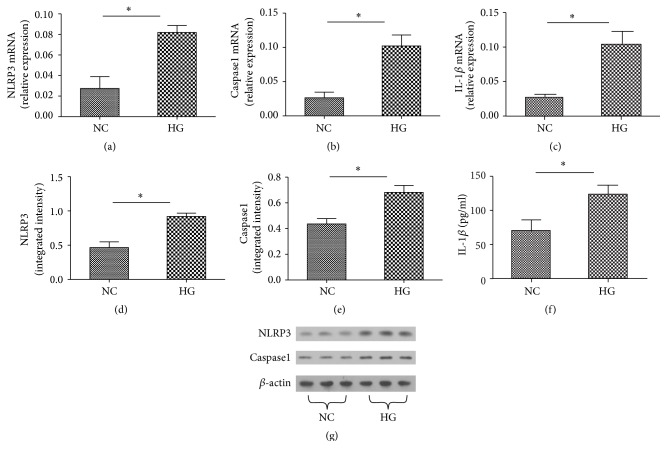
mRNA and protein expression of NLRP3, caspase1, and IL-1*β* with high glucose stimulation. RNA was measured by real-time PCR (a, b, and c) and proteins were measured by Western blot (d, e, and g) or Elisa (f), respectively. Values are mean ± SD; ^*∗*^*P* < 0.05.

Compared to normal control group, NLRP3, caspase1, and IL-1*β* were significantly increased at both the mRNA and protein levels when induced by 30 mmol/L glucose (*P* < 0.05) (Figures 2(a)–2(g)).

### 3.4. Effects of High Glucose on the Expression of Surface Markers on Macrophages

**Figure 3:**
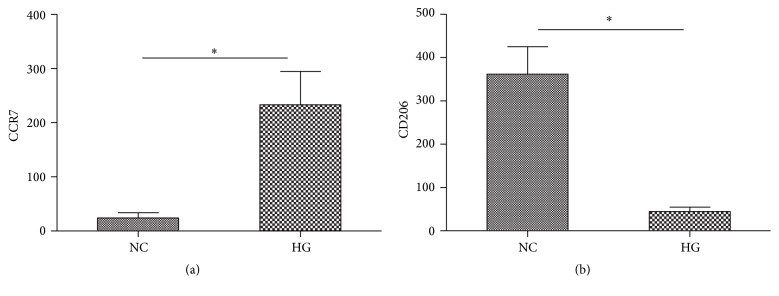
Cell surface CCR7 (a) and CD206 (b) expression in human macrophages exposed to high level of glucose. ^*∗*^*P* < 0.05.

M1 macrophages usually express a high level of cell surface markers such as CCR7, while M2 macrophages express a higher level of mannose receptor (CD206) [12]. We accessed the impacts of high glucose on the expressions of these surface markers by FCM. As expected, an expression of CCR7 was significantly upregulated on macrophages after high glucose stimulation (Figure 3(a)). In addition, the level of CD206 was lower when the macrophages have been exposed to high glucose concentrations (Figure 3(b)).

### 3.5. Inhibition of NLRP3 Decreases IL-1*β* Activity in High Glucose Induced Macrophages

**Figure 4:**
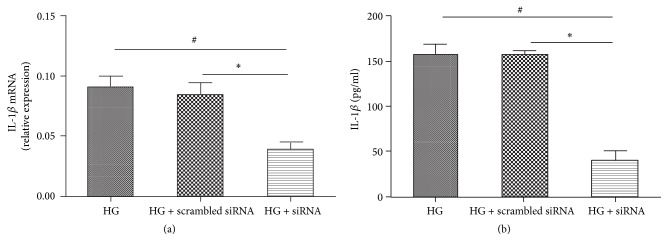
IL-1*β* expression related to NRLP3 in macrophages transfected with siNLRP3. Cells are either silenced with siNLRP3 or treated with a scrambled control RNA and then exposed to high glucose. RNA and protein expression of IL-1*β* were measured by real-time PCR (a) or Elisa (b), respectively. Values are mean ± SD; ^#^*P* < 0.05, ^*∗*^*P* < 0.05.

We knocked down NLRP3 levels by siRNA-mediated gene silencing. A scrambled siRNA was used as a control. Transfection with siNLRP3 was evidently effective in high glucose conditions, as reflected by decreased IL-1*β* expression at both the mRNA and protein levels (Figures 4(a) and 4(b)).

## 4. Discussion

An impaired diabetic wound healing process is a worldwide problem and a major cause of morbidity and mortality in diabetic patients [13]. It is typically associated with persistent inflammation response due to infiltration of immune cells and cytokines [14]. Several inflammatory cytokines are activated during the process of wound healing. An antagonistic relationship appears to exist within the wound microenvironment between the pathways of proinflammatory cytokines such as IL-1*β* and TNF-*α* and anti-inflammatory growth factors/cytokines/hormones, as in the case of TGF-*β*/IL-1ra//IL-10/E2. The role of this counterbalance in the pathogenesis of disturbed wound healing during diabetes remains to be fully elucidated [15].

In this study, we investigated the NLRP3 inflammasome in human diabetic wounds, and our results suggested the upregulation of NLRP3, caspase1, and IL-1*β* mRNA in human diabetic wounds. Consistent with this, significantly higher protein levels of NLRP3, caspase1, and IL-1*β* were also observed in diabetic wound group. Taken together, these data suggest that NLRP3 inflammation activation might be involved in the pathogenesis of wound healing impairment.

NLRP3 inflammasome is one of the largest as well as the most studied cytosolic inflammasomes, comprised of NLRP3, adapter molecule ASC, and procaspase1. During activation, procaspase1 is recruited to the NLRP3 inflammasome and cleaved to produce active caspase1 and then cleaves and activates the potent proinflammatory cytokines IL-1*β*. Considering a regulatory role of NLRP3 in inflammation and adaptive immune repose, its aberrant expression or function has been reported in many inflammatory diseases. Luo et al. [16] found NLRP3 may play an important role in the pathogenesis of diabetic cardiomyopathy, and silencing NLRP3 ameliorated cardiac remodeling and dysfunction. Chen et al. [17] showed that activation of the NLRP3 inflammasome regulates IL-1 family cytokine secretion and causes the development of tubulointerstitial inflammation in diabetic nephropathy. The chronic inflammation associated with obesity has been shown to contribute directly to the development of insulin resistance and ultimately type 2 diabetes and also affects diabetic microvascular complications [18]. As NLRP3 is involved in IL-1*β* processing, we also analyzed IL-1*β* release. In our study, both IL-1*β* mRNA and protein were upregulated in the wounds of diabetic patients, when compared with nondiabetic wounds.

Although the role of NLRP3 inflammasome activation in diabetes has been demonstrated, the molecular targets utilizing high glucose during NLRP3 inflammasome activation in macrophages are not well understood. The pathophysiological relevance of NLRP3 inflammasome in human diabetic wound was accessed in the cultured biopsies. In the diabetic wound, NLRP3 colocalized with macrophages. Moreover, our ex vivo experiments suggest the upregulation of NLRP3, caspase1, and IL-1*β* mRNA and protein in high glucose induced macrophages. This findings support a role for NLRP3 inflammasome in macrophages in human diabetic wound, linking high glucose accumulation and inflammation with the impaired wound healing.

In our study, we find that high glucose not only predominantly induces macrophages to secrete inflammatory cytokines but induces M1 polarization as evidenced by the expression of surface markers on macrophages. The expression of M1 surface marker CCR7 was upregulated, and the expression of M2 surface marker CD206 was low after high glucose treatment. Diabetic wounds are typically associated with a persistent inflammatory response that involves accumulation of macrophages. There is a switch from proinflammatory to prohealing macrophages phenotypes during normal wound healing and the diabetic wound environment impairs the switch to a healing-associated macrophages phenotype. IL-1*β* is a critical proinflammatory cytokine in the process of proinflammatory macrophage (M1) phenotype. The sustained production of IL-1*β* in the diabetic wound environment acts as a positive feedback loop to sustain the proinflammatory macrophage phenotype and blocks the induction of a healing-associated macrophage phenotype [19]. In the previous study, Mirza et al. [4] applied IL-1*β*-blocking antibody locally to wounds in vivo and found inhibiting IL-1*β* downregulates the proinflammatory macrophage phenotype and upregulates expression of prohealing factors in wounds of diabetic mice and improves healing of these wounds. In our study, high glucose stimulation can induce macrophages polarization into M1 phenotype partly through the NLRP3/IL-1*β* pathway and this might be one of the mechanisms responsible for the diabetic complications.

NLRP3 is unique in its ability to recognize molecular patterns associated with host-derived signals that are abundant in obese individuals, including excess ATP, glucose, and ROS [20]. The expression of NLRP3, caspase1, and IL-1*β* was significantly increased by high glucose concentration. When knocking down NLRP3 levels by siRNA-mediated gene silencing, a decreased expression of IL-1*β* in high glucose induced macrophages was noted. Song et al. [21] reported Mangiferin treatment attenuated the expressions of NLRP3 and reduced IL-1*β* production in the presence of high glucose. Caspase1 mediated cleavage is the limiting step for processing IL-1*β* into its secreted active forms [22]. Previous studies reported the activation of NLRP3/ASC/caspase1 inflammasome culminated in the production of IL-1*β* [23]. ASC act as the indispensable adaptor that connects NLRP3 and procaspase1 [24]. These clear evidences showed that IL-1*β* secretion is regulated by the NLRP3 inflammasome in high glucose induced macrophages.

## 5. Conclusion

In conclusion, the present study demonstrated a significant higher expression of NLRP3, caspase1, and secretion of IL-1*β* in the human diabetic wound and in high glucose induced macrophages, suggesting that NLRP3 inflammasome activation might contribute to the hyperinflammation in would healing. High glucose induced macrophages polarization into M1 phenotype was partly dependent on NLRP3/IL-1*β* activation. The results indicated that NLRP3 inflammation may be a novel target to treat the diabetic patients with chronic wound.

## Figures and Tables

**Table 1:** Clinical characteristics of diabetic patients and controls.

	Diabetic patients (*N* = 6)	Control (*N* = 6)	*P*
Age (years)	51.0 ± 11.4	43.7 ± 8.8	*P* > 0.05
Body mass (kg)	69.3 ± 9.3	65.5 ± 8.5	*P* > 0.05
Body mass index (kg/m2)	25.4 ± 2.5	23.7 ± 1.3	*P* > 0.05
Duration of diabetes (years)	6.7 ± 3.5	—	—
Insulin (*n*/*N*)	3/6	—	—
Antidiabetic drug (*n*/*N*)	3/6	—	—

**Table 2:** Laboratory data of diabetic patients and controls.

	Diabetic patients (*N* = 6)	Control (*N* = 6)	*P*
Fasting blood glucose (mmol/L)	8.7 ± 1.1	5.4 ± 0.5	*P* < 0.01
2 h blood glucose (mmol/L)	13.7 ± 2.2	6.5 ± 0.9	*P* < 0.01
HbA1c (%)	7.8 ± 1.2	5.2 ± 0.5	*P* = 0.01
LDL-C (mmol/L)	2.8 ± 0.2	2.5 ± 0.1	*P* > 0.05
SCr (*μ*mol/L)	73.7 ± 13.0	68.2 ± 8.8	*P* > 0.05
BUN (*μ*mol/L)	4.7 ± 0.9	3.8 ± 0.6	*P* > 0.05
GFR (ml/min)	98.4 ± 10.3	117.8 ± 33.3	*P* > 0.05

## References

[1] Wild S., Roglic G., Green A., Sicree R., King H. (2004). Global prevalence of diabetes: estimates for the year 2000 and projections for 2030. *Diabetes Care*.

[2] Marfella R., Sasso F. C., Rizzo M. R. (2012). Dipeptidyl peptidase 4 inhibition may facilitate healing of chronic foot ulcers in patients with type 2 diabetes. *Experimental Diabetes Research*.

[3] Ozaki E., Campbell M., Doyle S. L. (2015). Targeting the NLRP3 inflammasome in chronic inflammatory diseases: current perspectives. *Journal of Inflammation Research*.

[4] Mirza R. E., Fang M. M., Ennis W. J., Kohl T. J. (2013). Blocking interleukin-1*β* induces a healing-associated wound macrophage phenotype and improves healing in type 2 diabetes. *Diabetes*.

[5] Weinheimer-Haus E. M., Mirza R. E., Koh T. J. (2015). Nod-like receptor protein-3 inflammasome plays an important role during early stages of wound healing. *PLoS ONE*.

[6] Martinon F., Burns K., Tschopp J. (2002). The inflammasome: a molecular platform triggering activation of inflammatory caspases and processing of proIL-*β*. *Molecular Cell*.

[7] Kim J.-J., Jo E.-K. (2013). NLRP3 inflammasome and host protection against bacterial infection. *Journal of Korean Medical Science*.

[8] Blakytny R., Jude E. (2006). The molecular biology of chronic wounds and delayed healing in diabetes. *Diabetic Medicine*.

[9] Mcgettrick A. F., O'Neill L. A. J. (2013). NLRP3 and IL-1*β* in macrophages as critical regulators of metabolic diseases. *Diabetes, Obesity & Metabolism*.

[10] Bitto A., Altavilla D., Pizzino G. (2014). Inhibition of inflammasome activation improves the impaired pattern of healing in genetically diabetic mice. *British Journal of Pharmacology*.

[11] Mustoe T. A. Dermal ulcer healing: advances in understanding.

[12] Labonte A. C., Tosello-Trampont A.-C., Hahn Y. S. (2014). The role of macrophage polarization in infectious and inflammatory diseases. *Molecules and Cells*.

[13] Hunt S. D., Elg F. (2016). Clinical effectiveness of hemoglobin spray (Granulox ®) as adjunctive therapy in the treatment of chronic diabetic foot ulcers. *Diabetic Foot & Ankle*.

[14] Feng H., Gu J., Gou F. (2016). High Glucose and Lipopolysaccharide Prime NLRP3 Inflammasome via ROS/TXNIP Pathway in Mesangial Cells. *Journal of Diabetes Research*.

[15] Al-Mulla F., Leibovich S. J., Francis I. M., Bitar M. S. (2011). Impaired TGF-*β* signaling and a defect in resolution of inflammation contribute to delayed wound healing in a female rat model of type 2 diabetes. *Molecular BioSystems*.

[16] Luo B., Li B., Wang W. (2014). Rosuvastatin alleviates diabetic cardiomyopathy by inhibiting NLRP3 inflammasome and MAPK pathways in a type 2 diabetes rat model. *Cardiovascular Drugs and Therapy*.

[17] Chen S., Sheng C., Liu D. (2013). Enhancer of zeste homolog 2 is a negative regulator of mitochondria-mediated innate immune responses. *Journal of Immunology*.

[18] Klen J., Goričar K., Janež A., Dolžan V. (2015). NLRP3 inflammasome polymorphism and macrovascular complications in type 2 diabetes patients. *Journal of Diabetes Research*.

[19] Mirza R. E., Fang M. M., Weinheimer-Haus E. M., Ennis W. J., Koh T. J. (2014). Sustained inflammasome activity in macrophages impairs wound healing in type 2 diabetic humans and mice. *Diabetes*.

[20] Mori M. A., Bezy O., Kahn C. R. (2011). Metabolic syndrome: is Nlrp3 inflammasome a trigger or a target of insulin resistance?. *Circulation Research*.

[21] Song J., Li J., Hou F., Wang X., Liu B. (2015). Mangiferin inhibits endoplasmic reticulum stress-associated thioredoxin-interacting protein/NLRP3 inflammasome activation with regulation of AMPK in endothelial cells. *Metabolism: Clinical and Experimental*.

[22] Jourdan T., Godlewski G., Cinar R. (2013). Activation of the Nlrp3 inflammasome in infiltrating macrophages by endocannabinoids mediates beta cell loss in type 2 diabetes. *Nature Medicine*.

[23] Zheng S.-C., Zhu X.-X., Xue Y. (2015). Role of the NLRP3 inflammasome in the transient release of IL-1*β* induced by monosodium urate crystals in human fibroblast-like synoviocytes. *Journal of Inflammation*.

[24] Babelova A., Moreth K., Tsalastra-Greul W. (2009). Biglycan, a danger signal that activates the NLRP3 inflammasome via toll-like and P2X receptors. *Journal of Biological Chemistry*.

